# Supramolecular Study of the Interactions between Malvidin-3-*O*-Glucoside and Wine Phenolic Compounds: Influence on Color

**DOI:** 10.1021/acs.jafc.2c08502

**Published:** 2023-02-07

**Authors:** Bárbara Torres-Rochera, Elvira Manjón, Natércia F Brás, María Teresa Escribano-Bailón, Ignacio García-Estévez

**Affiliations:** † Grupo de Investigación en Polifenoles (GIP), Departamento de Química Analítica, Nutrición y Bromatología, Facultad de Farmacia, Universidad de Salamanca, Salamanca E37007, Spain; ‡ LAQV, REQUIMTE, Departamento de Química e Bioquímica, Faculdade de Ciências, Universidade do Porto, 4169-007 Porto, Portugal

**Keywords:** malvidin-3-*O*-glucoside, wine phenolic compounds, tristimulus colorimetry, ITC, MD

## Abstract

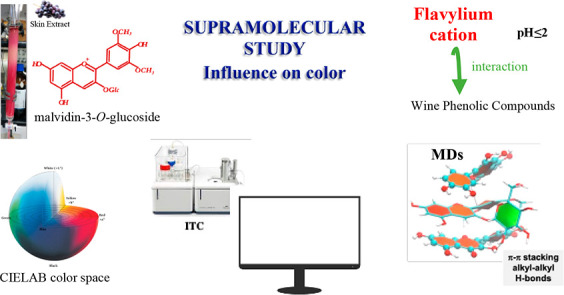

Supramolecular study of the interactions between the major wine anthocyanin, malvidin-3-*O*-glucoside (Mv3G) and different wine phenolic compounds (quercetin 3-*O*-β-glucopyranoside (QG), caffeic acid, (−)-epicatechin, (+)-catechin, and gallic acid) has been performed at two different molar ratios (1:1 and 1:2) in acidic medium where flavylium cation predominates (pH ≤ 2). Color variations have been evaluated by differential colorimetry using CIELAB color space. These studies have been complemented with isothermal titration calorimetry assays and molecular dynamics simulations. The color of Mv3G flavylium cation is modified by the interaction with QG toward more bluish and intense colors. Interaction constants between the anthocyanin and the different phenolic compounds were obtained, ranging from 9.72 × 10^8^ M^–1^ for QG to 1.50 × 10^2^ M^–1^ for catechin. Hydrophobic interactions and H-bonds are the main driving forces in the pigment/copigment aggregation, except for the interactions where caffeic acid is involved, in which hydrophobic interactions acquire greater preponderance.

## Introduction

Anthocyanins, which are phenolic compounds that belong to the flavonoid family (C6–C3–C6), are one of the most important pigments responsible for the red color in many fruits, vegetables, juice, jams, and red wines.^1,2^ Red wines contain different anthocyanins, with malvidin-3-*O*-glucoside being the most abundant one in young wines, which is usually found in concentrations around 300–500 mg/L.^3^ The red color of anthocyanins is due to the flavylium cation, which is the stable form at acidic pH (pH ≤ 2). However, anthocyanins have a high tendency to undergo alterations in their plane structure by different reactions depending on the pH of the medium, which make them relatively unstable in aqueous solutions.^4^ Hydration reaction, which leads to colorless hemiketal or acid–base equilibria that leads to blue purple quinoidal base, can take place when pH raises, thus decreasing the flavylium cation levels.^5^

The flavylium cation is protected from the nucleophilic attack of water due to copigmentation effect, which consists of a sandwich stacking between the anthocyanin and other organic molecules, known as copigments. This binding is due to noncovalent hydrophobic molecular associations, mainly π–π stacking interactions, between the aromatic nuclei of the colored forms of anthocyanins and copigments.^6^ As a result, the hydration reaction is reduced, which limits the formation of colorless compounds, with copigmentation being responsible for 30–50% of the coloration in red young wines.^3^ Likewise, self-association is another mechanism to stabilize the red color of the flavylium cation, since anthocyanins can act themselves as copigments, resulting in an increase in the color intensity.^3,7,8^

Among wine phenolic compounds, flavanoids and hydroxycinnamic acids seem to be the most important compounds that could act as anthocyanin copigments. It is well-known that flavylium cation interacts with flavanols, such as (−)-epicatechin and (+)-catechin, with the former one being a better copigment than the latter.^9^ Likewise, flavonols have shown a great capacity to act as copigments,^3,10,11^ since their pigment–copigment association constants are higher than those found in the flavanol–anthocyanin interactions. However, their quantity in wine is much lower than that of flavanols. With regards to other phenolic compounds, the hydroxycinnamic acids, specially caffeic acid, have also shown a strong copigmentation effect,^12,13^ whereas benzoic acids, like gallic acid, are generally less efficient as copigments.^3,14^

Copigmentation has been widely studied *via* spectrophotometric methods^6,15−17^ since anthocyanins absorb in the visible range due to their π-conjugated systems. Besides, color characterization in the CIELAB space by differential colorimetry provides a complete colorimetric interpretation of the anthocyanin–copigment interactions, leading to a knowledge about chemistry of the anthocyanin color. However, UV–vis spectroscopy does not allow to characterize the molecular interactions that govern the copigmentation process from a supramolecular point of view.

Isothermal titration calorimetry (ITC) is an interesting technique that allows for the determination of thermodynamic parameters (Δ*G*, *K*, Δ*H*, and Δ*S*), in order to study binding mechanisms in supramolecular systems. This technique makes possible to measure the changes of energy that occurs by reversible interactions,^18−20^ even when the interactions are weak or in low affinity systems.^21,22^ Likewise, molecular dynamics (MD) simulation allows for the estimation of binding energies values and the stability of the resulting complexes using model systems to better understand the interactions between different molecules, which makes MD simulations a very useful computational method that can be employed to complement experimental works.

Thus, the objective of this study was to analyze, for the first time, the interaction of the flavylium cation with different phenolic compounds already known as copigments from a supramolecular point of view. Also, this work aimed to establish relationships between the color changes expressed by the flavylium form of Mv3G and the strength of the interaction, the forces involved, and the characteristics of complexes formed. To do this, the interactions between Mv3G and different phenolic compounds (PC), namely, a flavonol (quercetin 3-*O*-β-glucopyranoside (QG)), two flavanols ((−)-epicatechin (E) and (+)-catechin (C)), an hydroxycinnamic acid (caffeic acid (CA)), and an hydroxybenzoic acid (gallic acid (GA)) were analyzed by using UV–vis spectroscopy, ITC, and MD simulations.

## Materials and Methods

### Chemicals

Quercetin 3-*O*-β-glucopyranoside (≥99%) was purchased from Cymit Quimica (Barcelona, Spain). (−)-Epicatechin (≥90%) and (+)-catechin hydrate (≥98%) were purchased from Sigma-Aldrich (St. Louis, MO). Caffeic acid (≥99%) was purchased from ACROS organics (Morris Plains, New Jersey) and gallic acid (≥98%) was purchased from Merck (Darmstadt, Germany). Ultrapure water was obtained from a Mili-Q Gradient water purification system (Millipore, Billerica, MA). The pigment malvidin-3-*O*-glucoside was isolated in the laboratory as is explained below.

### Isolation of Malvidin-3-*O*-Glucoside

Mv3G was isolated from skins of *Vitis vinifera* cv Tempranillo grapes. Grape skins were extracted by using acidic methanol (methanol/HCl 0.5 N; 95:5 *v/v*), as described in García-Estévez et al.^23^ In order to purify the Mv3G, the residue was loaded on a Sephadex LH-20 (Sigma-Aldrich, St. Louis, MO) column, previously conditioned with acidic water as eluent using aqueous HCl (0.1 M, pH 1.0) as described by García-Estévez et al. with minor modifications.^24^ Briefly, elution was carried out using the aqueous HCl solution, and the first fraction (*ca*. 20 mL) was collected, which corresponded to Mv3G. This process was repeated to obtain several fractions. The purity of these fractions was checked by HPLC–DAD-MS, and those showing purity higher than 95% were gathered and then freeze-dried to furnish a dark reddish-purple powder.

### Model Solutions

All model solutions were prepared at acidic medium (pH 1.1). The pigment concentration was the same in all cases (50 μM), which was selected to minimize self-association effect. Five solutions based on binary combinations of Mv3G with quercetin 3-*O*-β-glucopyranoside (Mv3G/QG), caffeic acid (Mv3G/CA), (−)-epicatechin (Mv3G/E), (+)-catechin hydrate (Mv3G/C), and gallic acid (Mv3G/GA) were prepared at two different pigment/phenolic compound (PC) molar ratios (1:1 and 1:2) to evaluate the effect of the PC concentration. A reference solution of the anthocyanin at the same pH was also prepared. All solutions were prepared in triplicate and stored in darkness at 25 °C for 2 h to reach equilibrium.

### Color Analysis

Absorption spectra (190–1100 nm) of the above solutions were recorded on a Hewlett-Packard UV–vis HP3853 spectrophotometer (Agilent Technologies, Waldbronn, Germany) at constant intervals (Δλ = 1 nm), using 10 mm path length quartz cells and acidic water (pH 1.1) as a reference. The analysis of color was studied from the visible spectra (380–770 nm) data, using a CIE 1964 standard observer (10 visual field) and a CIE standard illuminant D65 as references. The CIELAB color parameters (L*, a*, b*, C*_ab_, and *h*_ab_) were calculated using the software Cromalab (University of Sevilla, Sevilla, Spain).^25^

Color differences between the reference solution of the anthocyanin and the binary pigment:PC solutions (ΔE*_ab_) were also calculated using the CIELAB color difference equation: ΔE*_ab_ = [(ΔL*)^2^ + (Δa*)^2^ + (Δb*)^2^]^1/2^, where ΔL*, Δa*, and Δb* are the differences between the CIELAB parameters. The colorimetric effect of the interactions over the storage period was evaluated by differential colorimetry according to Gordillo et al.^16,17^

In addition, ΔL*, ΔC*_ab_, and *Δh*_ab_ absolute attributes were also calculated to evaluate the trend of color changes produced. The relative contribution of each color attribute for total color differences was calculated as follows:^16,17^
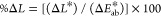

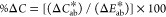

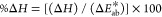
The Δ*H* value was deduced from Δ*H* = [ΔE*_ab_ – ((Δ*L*)^2^ + (Δ*C*)^2^)]^1/2^

### Isothermal Titration Calorimetry Assays

ITC experiments were carried out by using a MicroCal PEAQ-ITC system (Malvern, U.K.) in order to obtain the thermodynamic parameters associated with pigment/PC interactions. In all cases, the PC solutions were loaded into the injection syringe, whereas the pigment solution was placed into the 0.2 mL sample cell of the calorimeter, being the content of the sample cell constantly stirred at 750 rpm throughout the experiment. Each assay consisted on a sequence of 19 injections of 2 μL each one, with the time of the injection duration and the time between the successive injections set as 4 and 150 s, respectively. Interactions were studied at 298 K. All solutions were prepared at pH 1.1 at the following concentrations: 200 μM Mv3G/250 μM QG; 400 μM Mv3G/3.000 μM CA; 800 μM Mv3G/10.000 μM E; 1.000 μM Mv3G/10.000 μM C; and 1.000 μM Mv3G/10.000 μM GA. These concentrations were selected to achieve the saturation of the process and enough energy signal recorded. Blank experiments, where the sample cell was filled with acidic water (pH 1.1), were also conducted for each compound at all concentrations previously assayed. All experiments were performed in triplicate.

The software AFFINimeter (Software for Science Developments, Santiago de Compostela, Spain) was used for data treatment. This software allowed us, by using the stoichiometric equilibria model, to obtain the fitting curve (enthalpy change vs molar ratio), as well as the binding apparent constant (*K*), the Gibbs free energy (Δ*G*), the enthalpy change (Δ*H*), and the entropy component (−*T* Δ*S*).

### Molecular Modeling and Molecular Dynamics (MD) Simulations

The GaussView software^26^ was used to build the flavylium cation structure of the Mv3G and the different phenolic compounds (PC) assayed. Each system was composed by 5 Mv3G and 11 PC molecules (5:11 pigment/phenolic compound) to reproduce the conditions used in the previous experiments. All compounds were randomly positioned, with a minimum distance between them of 15 Å. An explicit solvation model with pre-equilibrated TIP3P water molecules was used, filling a truncated rectangular box with a minimum distance of 15 Å between the box faces and any atom of each system. The Amber 12 simulation package^27^ was used to carry out the optimizations and MD simulations. General AMBER force field (GAFF)^28^ parameters were assigned using the antechamber module with RESP charges^29^ at the HF/6-31G* level. Similar parameters for malvidin 3-*O*-glucoside, quercetin, caffeic acid, gallic acid, catechin, and epicatechin were used in previous studies.^20,30−32^ Each starting system was minimized and equilibrated for 100 ps, followed by 100 ns of production MD simulation. To increase the sampling, three replicates, starting from different initial velocities, were simulated for each Mv3G/PC system. The pressure and the temperature of the systems were controlled by using the Berendsen barostat and the Langevin thermostat.^33^ The SHAKE algorithm^34^ was employed to constrain the bond lengths involving hydrogen atoms. Periodic boundary conditions were considered. Nonbonded interaction pairs were calculated within 10 Å. Beyond that, Coulomb interactions were treated with the Particle-Mesh Ewald (PME) method,^35^ and vdW interactions were truncated. The MD trajectories were analyzed with the CPPTRAJ module^36^ of AMBER 12.0 simulations package, combined with the visual molecular dynamics (VMD 1.9.2) program for visualization, analysis, and image rendering.^37^

The prevalent Mv3G/PC (1:1 complex) of each system was used as starting geometry of a further MD simulation to better understand the interaction mode between the two molecules. The binding energy of each Mv3G/PC complex was determined using the molecular mechanics/Poisson–Boltzmann surface area (MM/PBSA) approach.^37,38^ A total of 100 structures of each MD simulation were used for the analysis. The results are shown as the relative enthalpic binding energies (ΔΔ*H*_binding_) with respect to the most stable geometry.

### Statistical Analysis

The statistical significance of the differences between the results from the colorimetric analysis were evaluated by one-way analysis of variance (ANOVA) and posthoc Tukey test using the software packing for Windows IBM SPSS 26 (SPSS, Inc. Chicago, IL). Differences were considered statistically significant at *p* < 0.05.

## Results and Discussion

### Color Assays

Binary solutions containing Mv3G and PC at two different molar ratios (1:1; 1:2) were prepared at pH 1.1 to evaluate the effect of the Mv3G/PC interaction on the color of the anthocyanin flavylium cation. At this pH, the colored flavylium cation predominates and the determination of the CIELAB parameters (L*, a*, b*, C*_ab_, and *h*_ab_) would allow us to evaluate possible changes of color due to these interactions in relation to the pure anthocyanin.

Table 1 shows the CIELAB color parameters (L*, a*, b*, C*_ab_, and *h*_ab_) determined for the Mv3G control solution and for the Mv3G/PC solutions after 2 h of preparation. L* value corresponds to the lightness of the solutions and varies from 0 to 100, (black and white color, respectively); a* and b* are chromaticity scalar coordinates where a* axis is labeled from red (+a*) to green (−a*) color for positive and negative values, respectively, and b* axis from yellow (+b*) to blue (−b*). From the chromaticity, other coordinates can be defined, specifically chroma (C*_ab_), which is a quantitative attribute of color, and the hue angle (*h*_ab_), which is a qualitative attribute.

**Table 1:** CIELAB Color Parameters (L*, a*, b*, C*_ab_, and *h*_ab_) as well as ΔE*_ab_ and λ_max_ Determined for the Anthocyanin and Each Pigment/PC Solution at Two Different Molar Ratios (1:1, 1:2)^a^

	L*	a*	b*	C*_ab_	*h*_ab_	ΔE*_ab_	λ_max_
Mv3G control	78.5 ± 0.3	46.0 ± 0.6	1.7 ± 0.2	46.1 ± 0.6	2.1 ± 0.3		520
Mv3G/QG	78.0 ± 0.2	45.3 ± 0.2	–1.6 ± 0.2*	45.4 ± 0.2	–2.1 ± 0.2*	3.41 ± 0.15 ^a^	521
Mv3G/QG_2_	77.6 ± 0.1*	44.8 ± 0.2*	–4.2 ± 0.1**	45.0 ± 0.2*	–5.4 ± 0.2**	6.11 ± 0.17 ^a^	523
Mv3G/CA	79.0 ± 0.4	45.6 ± 0.6	1.3 ± 0.2	45.6 ± 0.6	1.6 ± 0.2	0.84 ± 0.62 ^b^	520
Mv3G/CA_2_	79.1 ± 0.5	45.8 ± 1.2	1.1 ± 0.4	45.8 ± 1.2	1.3 ± 0.5	1.33 ± 0.60 ^bc^	519
Mv3G/E	79.2 ± 0.2*	44.9 ± 0.4	1.2 ± 0.1	44.9 ± 0.4	1.6 ± 0.2	1.36 ± 0.41 ^b^	519
Mv3G/E_2_	79.1 ± 0.2*	45.4 ± 0.9	1.4 ± 0.2	45.5 ± 0.9	1.8 ± 0.2	1.09 ± 0.39 ^bc^	520
Mv3G/C	79.1 ± 0.6	45.9 ± 1.4	1.8 ± 0.4	46.0 ± 1.4	2.2 ± 0.4	1.32 ± 0.64 ^b^	520
Mv3G/C_2_	79.1 ± 0.2	46.0 ± 0.3	1.7 ± 0.1	46.0 ± 0.3	2.1 ± 0.2	0.67 ± 0.20 ^c^	519
Mv3G/GA	79.2 ± 0.3*	46.3 ± 1.0	1.8 ± 0.4	46.4 ± 1.0	2.3 ± 0.4	1.14 ± 0.38 ^b^	520
Mv3G/GA_2_	79.5 ± 0.2*	44.7 ± 0.4	1.3 ± 0.0	44.7 ± 0.4	1.7 ± 0.0	1.72 ± 0.38 ^b^	520

a An asterisk within each column indicates statistical differences between that solution and the Mv3G control. Two asterisks within each column indicates statistical differences between the two molar ratios assayed for each PC (*p* < 0.05). Different letters within ΔE*_ab_ column indicates statistical differences (*p* < 0.05).

It can be observed that L* values were higher for the Mv3G/CA, Mv3G/E, Mv3G/C, and Mv3G/GA solutions than that found in the Mv3G control solution at both molar ratios, suggesting a clearing effect with significant differences in the case of Mv3G/E and Mv3G/GA solutions (Table 1). However, Mv3G/QG solution showed the lowest L* values, denoting a darkest color, especially in the ratio 1:2, which showed significant differences with respect to Mv3G control (Table 1). Regarding a*, b*, C*_ab_, and *h*_ab_ parameters, there were only considerable differences between the color of Mv3G/QG solutions and the Mv3G control. As for the a* parameter, it took positive values, which can be related to the reddish color area, only showing significant differences (*p* < 0.05) between the anthocyanin and the Mv3G/QG_2_ solution. Changes were also observed for b* parameter, where Mv3G/QG and Mv3G/QG_2_ interactions resulted in the smallest and the most negative values of b*, showing color changes that denoted a more bluish color. More negative values were obtained for the ratio 1:2, which means that the flavylium cation undergoes a stronger color shift toward bluish colors as the QG concentration increases (Table 1).

C*_ab_ values were slightly lower in the solutions containing phenolic compounds than in the Mv3G control solution, although differences were significant only when compared to the solution containing QG in a ratio 1:2 (Table 1). Values for C*_ab_ were in concordance with a* parameters. Thus, it seems that color purity slightly decreases after the addition of the different phenolic compounds. The *h*_ab_ values followed the same trend that b* parameter, showing significant differences only in the solutions containing QG, where negative values were determined at both ratios (Table 1).

In summary, the addition of QG to the Mv3G solutions at pH 1.1 had the highest effect on the color parameters studied. L*, b*, and *h*_ab_ values diminished, denoting a darkest and more bluish color in the Mv3G solutions in the presence of QG. These could mean that the interaction between the flavylium cation of Mv3G and the aromatic rings of QG extends the conjugation through all the bimolecular system. It is known that lengthen a conjugated system with multiple bonds in a molecule leads to shift the absorption to longer wavelengths.^39^ In fact, the λ_max_ for QG solutions changed from 520 (Mv3G control solution) to 523 nm in the case of Mv3G/QG_2_ (Table 1), which means a bathochromic shift corresponding to a color change from red to more bluish. A bathochromic shift has also been reported in solutions containing malvidin 3,5-di-*O*-β-d-glucoside and 7-*O*-sulfoquercetin at pH 1.^3^

As for the other PC assayed, only the L* parameter showed differences and it moved toward higher values, denoting an evolution to the solutions toward achromatic colors, which could indicate that, due to the interaction, the complex involving the flavylium cation and these PC would show lower molar absorption coefficients than the free flavylium form.

To assess if the changes observed in color parameters were high enough to allow the discrimination by the human eye, color differences between control (Mv3G) and Mv3G/PC solutions were calculated. It is generally accepted that values of ΔE*_ab_ higher than three units are visually detectable.^40^ The color differences (ΔE*_ab_) determined here (Table 1) were not visually distinguished after the addition of the phenolic compounds except in the case of Mv3G/QG solutions, which showed values exceeding three units, which were more perceptible in the ratio 1:2 (6.11 units). The relative contributions of lightness (%Δ*L*), chroma (%Δ*C*), and hue (%Δ*H*) to color differences were also calculated for better analyzing the trend of the changes in the color attributes.^16^ The results showed quantitative and qualitative changes in color (Figure S1 in the Supporting Information). At the studied pH, a clear predominance of the qualitative contribution to the absolute difference of color is evidenced, with significantly higher contribution of hue %Δ*H* (93.1% and 94.3% for Mv3G/QG and Mv3G/QG_2_, respectively) with respect to lightness %Δ*L* or chroma %Δ*C* (between *ca*. 2.5–4.5%).

Hence, in our study, results pointed out that the color of flavylium cation solutions (pH 1) can be modified with the presence of QG, but not with C, E, CA, or GA. These observations lead to two possible alternatives: (i) the flavylium cation only interacts with QG (and not with C, E, CA, or GA; which would be against the current knowledge about the copigmentation effect) or (ii) the QG structure allows for interactions that change the Mv3G/QG electronic environment in a sensitive color way, but it does not occur in the Mv3G/C, Mv3G/E, Mv3G/CA, or Mv3G/GA interactions.

### Isothermal Titration Calorimetry

Isothermal titration calorimetry (ITC) has been employed to study the interactions between anthocyanin and the different phenolic compounds, since it is a successfully demonstrated technique for the characterization of molecular interactions.^20,41−45^ ITC experiments allowed us to obtain the thermodynamic parameters, i.e., free Gibbs energy change (Δ*G*), apparent binding constant (*K*), enthalpy variation (Δ*H*), and entropy variation (Δ*S*) of each anthocyanin/PC interaction that in turn allowed us to obtain the driving forces involved in them (Table 2).

**Table 2:** Thermodynamic Parameters Determined by ITC for each Interaction Mv3G/PC, at pH 1.1

	Δ*G* (cal·mol^–1^)	*K* (M^–1^)	Δ*H* (cal·mol^–1^)	–T·Δ*S* (cal·mol^–1^)
Mv3G/QG	–1.22 × 10^4^	9.72 × 10^8^	–4.01 × 10^3^	–8.23 × 10^3^
Mv3G/QG_2_	–2.44 × 10^4^	8.40 × 10^17^	–1.36 × 10^2^	–2.43 × 10^4^
Mv3G/CA	–5.59 × 10^3^	1.27 × 10^4^	–1.39 × 10^1^	–5.58 × 10^3^
Mv3G/CA_2_	–1.03 × 10^4^	3.85 × 10^7^	2.74 × 10^2^	–1.06 × 10^4^
Mv3G/E	–4.50 × 10^3^	2.01 × 10^3^	–2.56 × 10^2^	–4.24 × 10^3^
Mv3G/E_2_	–8.96 × 10^3^	3.78 × 10^6^	–1.50 × 10^2^	–8.81 × 10^3^
Mv3G/C	–2.96 × 10^3^	1.50 × 10^2^	–2.65 × 10^3^	–3.19 × 10^2^
Mv3G/C_2_	–5.88 × 10^3^	2.08 × 10^4^	–3.16 × 10^3^	–2.72 × 10^3^
Mv3G/GA	–3.09 × 10^3^	1.86 × 10^2^	–1.19 × 10^3^	–1.90 × 10^3^
Mv3G/GA_2_	–6.42 × 10^3^	5.15 × 10^4^	–1.21 × 10^3^	–5.21 × 10^3^

As can be seen in Table 2, all the Δ*G* values are negative, which indicates that spontaneous interactions are taking place in all the systems studied, and therefore, all PC studied interact with the flavylium cation of Mv3G. It is worth noting that the most negative value of Δ*G* is found in the samples containing Mv3G and QG, which suggests that, from the phenolic compounds studied, QG could be the one with the highest affinity for Mv3G. This is corroborated by the values of the affinity constant (*K*), which were notably higher for QG than for the other PC. According to *K* values, the affinity of Mv3G for the studied phenolic compounds was QG > CA > E > GA > C. These results are in agreement with those published by Zhao et al.,^46^ who reported that the binding capacities of these phenolic compounds follow similar order than the reported herein, thus indicating that ITC results can be compared to those obtained by colorimetry in terms of affinity.

Differences were found for the affinity of Mv3G toward the two phenolic acids studied: the *K* values for CA were higher than those for GA for the two analyzed ratios. As for flavanols, the *K* values suggest that E had a greater affinity toward Mv3G than C.

Regarding the type of forces involved, when the Δ*H* values are positive and −T·Δ*S* are negative, hydrophobic interactions are the main forces, whereas there is a hydrogen bonding prevalence when the Δ*H* data is negative and −T·Δ*S* has positive values. Both forces, hydrophobic interactions and hydrogen bonds (H-bonds), would take place when both Δ*H* and −T·Δ*S* are negative.^44^ On the basis of the results obtained, in general, the interactions involve both type of forces, H-bonds and hydrophobic bonds, with a slight prevalence of the latter ones.

### Molecular Dynamics (MD) Simulations

MD simulations were also used to assess the interaction between the flavylium form of the pigment Mv3G and the PC assayed. The complexes formed were assessed by a cluster analysis of each MD simulation, in which all geometries were collected using the root-mean square deviation of all heavy atoms of the Mv3G as metric (as they are the common molecules of all systems). Only the geometries with frequency higher than 5% along the MD simulation were considered for further analysis.

Table 3 shows the number and nature of the complexes 1:1 to 1:4 and 2:1 to 2:4 pigment/PC obtained from all MD simulations. The larger number of 1:1 and 1:2 pigment/PC aggregates formed suggest a tendency of these molecules to build the stacking complexes. In addition, the formation of PC/PC complexes were observed in all simulations (mainly in the systems with the CA molecules, see Table S1 of the Supporting Information (SI)). Regarding the Mv3G/Mv3G aggregates, they were formed in the simulations with the GA and E, less frequent in the systems with the CA and C, and inexistent in the simulations with the quercetin molecules (see Table S1 of the Supporting Information). This could mean preferential interaction between the Mv3G and the PC than between two molecules of the pigment (autoassociation).

**Table 3:** [Mv3G]n/[PC]n Complexes Formed Throughout the MD Simulations (geometries with a frequency >5%)

pigment/PC	1:1	1:2	1:3	1:4	2:1	2:2	2:3	2:4
Mv3G/caffeic acid	7	6	1	0	2	2	1	1
Mv3G/gallic acid	5	6	3	2	1	1	3	2
Mv3G/catechin	7	10	1	0	0	3	2	2
Mv3G/epicatechin	6	9	2	2	1	1	2	0
Mv3G/quercetin	4	2	3	2	1	0	1	1

**Figure 1:**
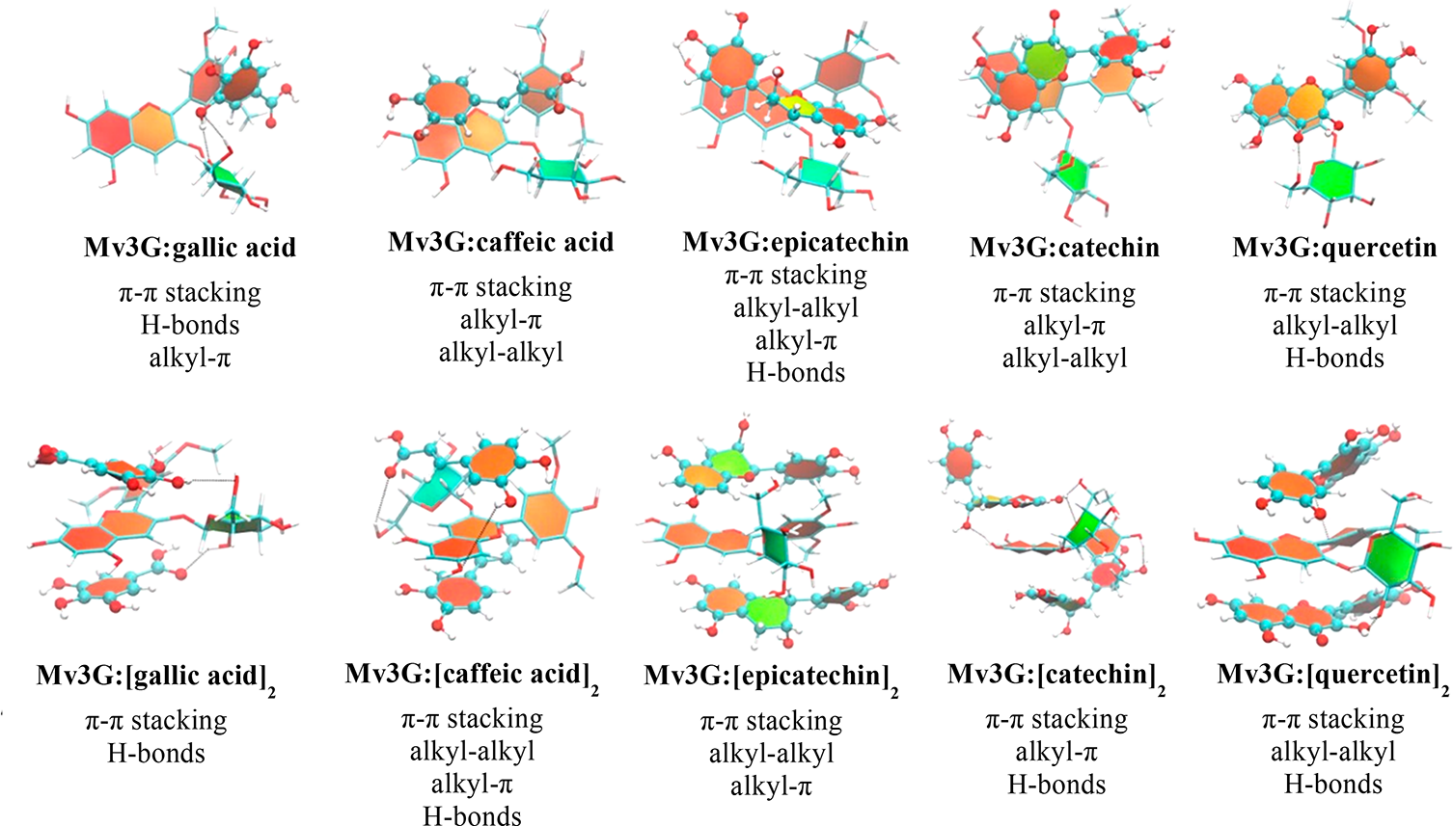
Representation of the most frequent 1:1 complexes pose using the Mv3G as pigment and the GA, CA, C, E, and QG as PC molecules. Mv3G is represented with sticks, while the PC are depicted in balls-and-sticks. All molecules are colored by atom type, with the aromatic and nonaromatic rings of the compounds being colored in orangish and green, respectively. Some H-bonds are also indicated as dashed lines.

In addition, MD simulations with 1:1 copigmentation complexes were carried out to assess the binding modes of the various PC to the Mv3G. Table 4 displays the binding energies of each Mv3G/PC complex, while Figure 1 illustrates the representative structure of the most frequent binding pose throughout each MD simulation. The main intermolecular interactions of each complex were also evidenced.

**Table 4:** Binding Energy Values (cal/mol) for the Mv3G/PC Complexes of All Systems^a^

PC	van der Waals	electrostatic	polar solvation	nonpolar solvation	Δ*H*_binding_
caffeic acid	(−11.09 ± 0.23) × 10^3^	(−0.74 ± 0.13) × 10^3^	(2.33 ± 0.11) × 10^3^	(4.04 ± 0.16) × 10^3^	(−5.47 ± 0.19) × 10^3^
gallic acid	(−10.76 ± 0.15) × 10^3^	(−0.91 ± 0.08) × 10^3^	(2.34 ± 0.08) × 10^3^	(3.83 ± 0.11) × 10^3^	(−5.50 ± 0.15) × 10^3^
catechin	(−16.12 ± 0.23) × 10^3^	(−1.51 ± 0.12) × 10^3^	(3.36 ± 0.10) × 10^3^	(5.66 ± 0.14) × 10^3^	(−8.61 ± 0.22) × 10^3^
epicatechin	(−17.69 ± 0.19) × 10^3^	(−1.29 ± 0.12) × 10^3^	(3.20 ± 0.10) × 10^3^	(5.86 ± 0.12) × 10^3^	(−9.92 ± 0.18) × 10^3^
quercetin	(−20.58 ± 0.19) × 10^3^	(−1.07 ± 0.09) × 10^3^	(3.45 ± 0.07) × 10^3^	(6.73 ± 0.09) × 10^3^	(−11.46 ± 0.19) × 10^3^

a Energy values are in cal/mol.

Concerning the enthalpic binding energies (Table 4), the highest absolute value was found for quercetin. Then, the affinity order to the Mv3G of the other PC molecules assayed was E ≈ C > GA ≈ CA. The presence of more than one aromatic ring in quercetin, epicatechin, and catechin may explain their higher binding affinity and highlights the importance of the π stacking interactions. This computed ranking is rather in line with the ITC results, in which quercetin was by far the compound with the highest affinity to Mv3G. As in ITC results, this could explain the colorimetric studies, in which quercetin caused significant color differences with respect to the control. It was also confirmed that the van der Waals forces were the main contributors to the binding energies (Table 4). Besides, the analysis of the intermolecular interactions of all complexes indicated the nonpolar and dispersive contacts as the main driving forces for the interaction of the five PC. In particular, π–π stacking between the aromatic rings of all polyphenolic molecules as well as the alkyl–alkyl interactions involving the methyl groups of Mv3G are present in all pigment/PC complexes. The exception was found in the complexes with gallic acid that did not make pronounced alkyl–alkyl contacts (Figure 1). Overall, this data agrees with other copigmentation studies involving anthocyanins, in which the π–π stacking and hydrophobic bonds have clearly been identified as driving forces of copigmentation.^3^ For example, previous MD simulations also pointed out the π–π stacking and van der Waals contacts as essential to drive the copigmentation between the large planar surfaces of the pigments (Mv3G or the malvidin-3,5-diglucoside) and copigments such as vinylcatechin dimers and catechin derivatives.^47,48^

Additionally, the MD results indicated that alkyl−π interactions were also relevant for the complexes with the caffeic acid, catechin, and epicatechin molecules (Figure 1). The CHCH moiety between the ring and the carboxylic group of CA promotes both alkyl–alkyl and alkyl−π contacts, while the B ring of C and E interacts with the glucose unit of the Mv3G. This type of interaction was not observed in the complexes involving quercetin, which mainly interact with the Mv3G by aromatic stacking involving their A, C, and B rings. Its double bond between C2–C3 reduces the B ring degrees of freedom, and subsequently, it sterically favors the π–π stacking. Furthermore, the negative quadrupole of the A ring would be reduced by the electron-withdrawing group in C-4, favoring sandwich and parallel displaced conformations over T-shaped configuration,^49^ as can be observed in Figure 1. This would allow for extending a highly conjugated π-bonding system that changes the visible light absorption of the system to longer wavelengths and that explains the bluish of the cation flavylium solutions evidenced in the colorimetric studies.

H-bonds are also important contacts for the interaction ability of these molecules. These hydrophilic interactions are noteworthy and short (lengths of 1.9–2.5 Å) in the complexes with gallic acid, highlighting the importance and subsequent contribution of the vicinal hydroxyl groups of its aromatic ring to the interaction ability.

Thus, the interaction binding mode obtained here for the Mv3G/C complex was similar to the three more favorable orientations described by Nave et al. for the 3-*O*-methylmalvidin/catechin complex.^50^ In addition, the binding mode for the malvidin 3-*O*-glucoside/quercetin complex was quite similar to the one depicted by a recent conformational quantum mechanics study. Like us, these authors also pointed out that the intermolecular ring-stacking and H-bonds play an important role in the malvidin 3-*O*-glucoside/quercetin stabilization.^51^

As conclusion remarks, the results show that, among the five PC studied in this work, the more stable interaction and the highest binding affinity was found between the flavylium cation and QG. The color of Mv3G solutions at acidic pH changed to darker colors and to bluish hues due to the presence of QG in the solutions, which is mainly related to a bathochromic shift in the flavylium band, more important when the QG concentration is increased. As for the other PC assayed, their effect on the color of flavylium form of Mv3G is less noticeable, just affecting the lightness of the solutions in the case of E and GA. The studies by ITC, which were in accordance with the results obtained in the MD simulations, pointed out that, among the PC assayed, the strongest interaction occurs between Mv3G and QG. This may be the reason why the presence of QG in the solutions provided the most important color differences. It was also observed that E has major binding affinity toward Mv3G than C, which could explain why significant differences of lightness regarding Mv3G control were just observed due to the presence of E. With regards to the type of forces, hydrophobic interactions and H-bonds are involved in all pigment/PC aggregates except for Mv3G/CA_2_, which is dominated by hydrophobic interactions.

## Supporting Information Available

Figure of relative contribution of lightness (%Δ*L*), chroma (%Δ*C*), and hue (%Δ*H*) to the total color differences for each pigment/copigment at molar ratio 1:1 and 1:2 and table of [Mv3G]n:[PC]n complexes formed throughout the MD simulations (geometries with a frequency >5%) (PDF)
