# Supplementary material for: Supramolecular Study of the Interactions between Malvidin-3-O-Glucoside and Wine Phenolic Compounds: Influence on Color
Source: J Agric Food Chem. 2023 Feb 7;72(4):1894–901. doi: 10.1021/acs.jafc.2c08502 (PMC10835720; doi:10.1021/acs.jafc.2c08502)
Supplement: Supplementary file 1 — jf2c08502_si_001.pdf [file jf2c08502_si_001.pdf]

Supramolecular study of the interactions between malvidin-3-*O*-glucoside and wine phenolic compounds. Influence on color

Bárbara Torres-Rochera[a], Elvira Manjón[a], Natércia F Brás[b], María Teresa Escribano-Bailón[a,*], Ignacio García-Estévez[a]

[a] Grupo de Investigación en Polifenoles (GIP), Departamento de Química Analítica, Nutrición y Bromatología, Facultad de Farmacia, Universidad de Salamanca, Salamanca E37007, España

[b] LAQV, REQUIMTE, Departamento de Química e Bioquímica, Faculdade de Ciências, Universidade do Porto, 4169-007 Porto, Portugal

*** Corresponding author:** María Teresa Escribano-Bailón.

E-mail address: escriban@usal.es

Telephone: +34 677596272 / +34 923 294537

E-mail addresses: barbara.torres@usal.es (B.T.R.) elvira87@usal.es (E.M.); nbras@fc.up.pt (N.F.B.); igarest@usal.es (I.G.E.)

Figure S1. Relative contribution of lightness (%Δ*L*), chroma (%Δ*C*) and hue (%Δ*H*) to the total color differences for each pigment/copigment at molar ratio 1:1 and 1:2.

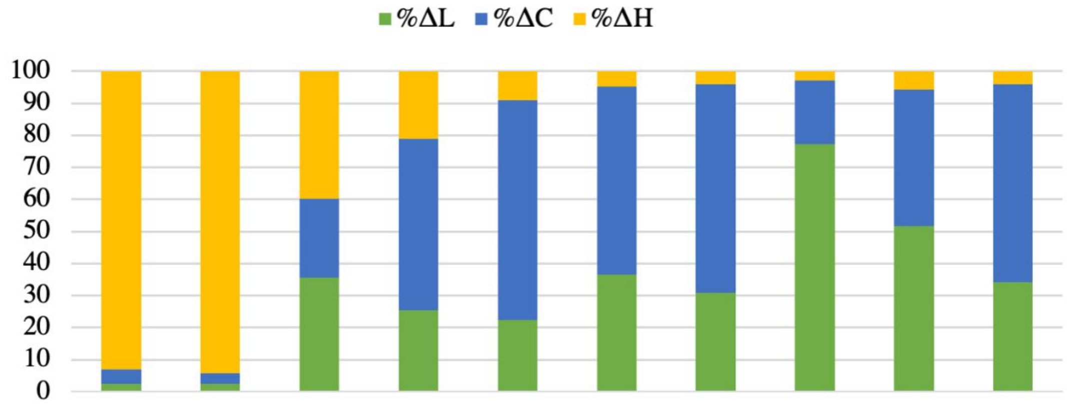

	Mv3G:QG	Mv3G:QG$_2$	Mv3G:CA	Mv3G:CA$_2$	Mv3G:E	Mv3G:E$_2$	Mv3G:C	Mv3G:C$_2$	Mv3G:GA	Mv3G:GA$_2$
%ΔH	93.09	94.27	39.87	21.07	8.94	4.82	4.13	2.78	5.62	3.98
%ΔC	4.46	3.18	24.51	53.44	68.63	58.68	65.13	19.87	42.78	61.78
%ΔL	2.45	2.55	35.62	25.49	22.44	36.50	30.74	77.35	51.60	34.24

Table S1. [Mv3G]n:[PC]n complexes formed throughout the MD simulations (geometries with a frequency >5%).

Complex	**2:5**	**2:6**	**2:7**	**3:3**	**3:4**	**3:5**	**3:7**	**3:8**	**3:9**	**4:2**	**4:4**	**4:8**	**5:8**	**PC:PC**	**Mv3G:Mv3G**
caffeic acid	1	0	0	0	1	0	0	0	0	0	0	1	1	7	No
gallic acid	0	1	0	0	0	0	0	0	0	1	0	0	0	2	Yes
catechin	2	1	0	0	1	1	0	0	0	0	0	0	0	3	No
epicatechin	1	1	0	0	0	2	0	0	0	0	0	0	0	3	Yes
quercetin	1	0	1	2	0	0	1	1	1	0	1	0	0	3	No
